# A Feasibility Study on the Use of the Method of Loci for Improving Episodic Memory Performance in Schizophrenia and Non-clinical Subjects

**DOI:** 10.3389/fpsyg.2021.612681

**Published:** 2021-02-05

**Authors:** Ana Elisa Sousa, Yacine Mahdid, Mathieu Brodeur, Martin Lepage

**Affiliations:** ^1^Comprehensive Research Into Schizophrenia and Psychosis (CRISP) Group, Integrated Program in Neurosciences, Douglas Mental Health University Institute, McGill University, Montréal, QC, Canada; ^2^Integrated Program in Neurosciences, McGill University, Montréal, QC, Canada; ^3^Douglas Mental Health University Institute, McGill University, Montréal, QC, Canada; ^4^Comprehensive Research Into Schizophrenia and Psychosis (CRISP) Group, Department of Psychiatry, Douglas Mental Health University Institute, McGill University, Montréal, QC, Canada

**Keywords:** psychosis, schizophrenia, method of loci, episodic memory, cognitive remediation

## Abstract

We investigated the feasibility of a short intervention using the Method of Loci (MoL), a well-known visuospatial mnemonic, to improve episodic memory recall performance in schizophrenia. The MoL training protocol comprised encoding and recall of two lists of items (words and images), a training session and practice with MoL. Then, participants had the opportunity to put into practice the newly learned MoL and were instructed to encode and recall two new lists of items using. This approach was first validated with healthy individuals (*N* = 71). Subsequently, five individuals with schizophrenia completed the protocol. Improvement in healthy individuals was observed for the word list (Wilcoxon effect size *r* = 0.15). No significant memory improvement was denoted in the schizophrenia group, possibly due to participants' difficulties using the method efficiently and due to fatigue. The MoL seems to require episodic memory, working memory monitoring and executive functions, making it suboptimal for a population with impairments in all those domains. Future research should examine the use of other strategies, better suited for individuals with cognitive impairments like those found in schizophrenia.

## Introduction

Episodic memory, one's ability to remember past events (Tulving, 1972), is severely impaired in schizophrenia (Feldmann et al., 2002; Berna et al., 2016), and such deficits are associated with compromised daily living activities and poor psychosocial functioning (Lepage et al., 2014). People with schizophrenia fail to spontaneously generate efficient strategies that improve memory recall (Iddon et al., 1998). The use of encoding strategies (e.g., forming a story, mental imagery, or using categories to link words) in non-clinical individuals often results in a richer memory-trace that subsequently increases recollection when compared to thinking about the word's visual features or using repetition (Bower, 1970; Tulving, 1972; Craik, 2002). When explicitly instructed to use similar strategies, individuals with schizophrenia can significantly improve their episodic memory performance (McClain, 1983; Ragland et al., 2003; Bonner-Jackson et al., 2005; Bonner-Jackson and Barch, 2011; Guimond and Lepage, 2016).

**Figure 1:**
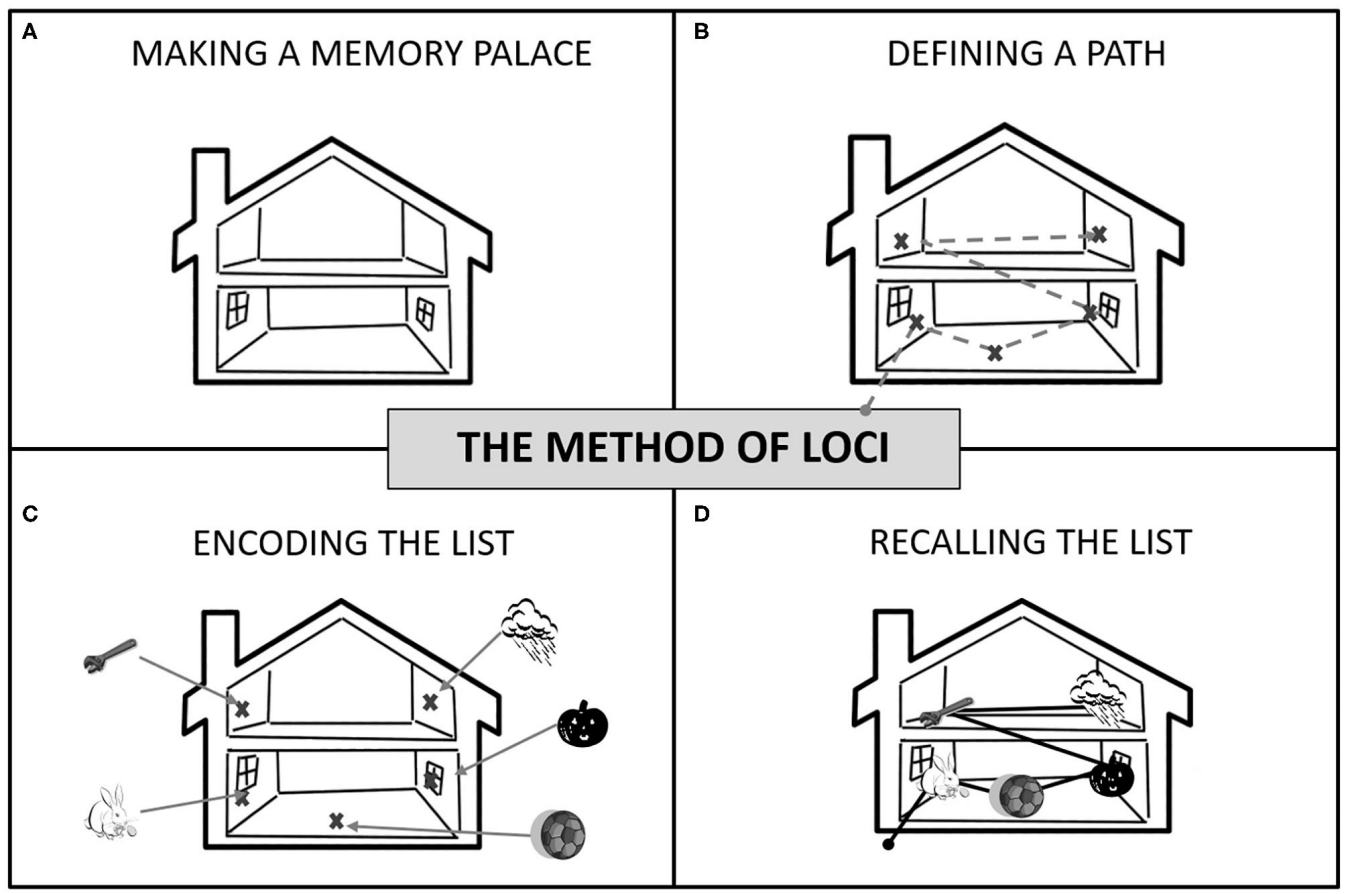
How to use the Method of Loci: **(A)** Make a memory palace by choosing a familiar building, route, or space (e.g., your home or the walk to the bus station); **(B)** Define a fixed route using places (your “*loci*”) as stopping points. If a house, use different rooms and furniture, if a path, use landmarks. You need as many *loci* as the number of items to remember. **(C)** To encode the list, mentally walk through your route. At each stop, place each item in a *locus* in the order they need to be memorized (i.e., the list order). Associations between loci and items must be vivid (e.g., pumpkin smashed into the window). **(D)** To recall, take a mental walk through your memory palace following the same route and “recover” items from each *locus* in the right order.

The Method of Loci (MoL) is a visuospatial mnemonic strategy that takes advantage of mental visualization and manipulation to facilitate encoding and recall of faces, digits, and word lists (Bower, 1970). MoL consists of mentally visualizing several previously selected and specific locations (loci) inspired in one's environment (i.e., one's home or neighborhood), within which to-be-memorized items are “placed” using mental manipulation. During recall, one has to mentally traverse their “memory palace” (a unique set of loci previously conceived for memorization) and recover the objects placed in each place (Bower, 1970). Figure 1 illustrates the different steps involved in this strategy. MoL was first attributed to the Greek poet Simonides of Ceos in 477 BC (Yates, 1992) and is widely used during memory competitions that require remembering a large amount of information, quickly and in order (e.g., World Memory Championship (Maguire et al., 2003).

MoL efficacy has been established in healthy participants (Bower, 1970; Carlson et al., 1976; Rose and Yesavage, 1983; Yesavage and Rose, 1984; Legge et al., 2012; McCabe, 2015), older adults (Rose and Yesavage, 1983; Yesavage and Rose, 1984; Anschutz et al., 1985; Gross et al., 2014), and even in individuals with depression (Dalgleish et al., 2013). Despite being one of the oldest and most popular mnemonics currently in use, to our knowledge, no study to date has examined whether people with schizophrenia can use and benefit from MoL (see Appendix for details). To investigate the acceptability and feasibility of MoL for improving episodic memory in schizophrenia, we developed a brief intervention involving free recall of two lists (concrete words or photos of single objects) before and after learning MoL. The intervention was first tested on a large sample of non-clinical participants to confirm the efficacy and then tested in individuals with schizophrenia. We expect that non-clinical participants will benefit from the intervention. Individuals with schizophrenia should also improve their memory performance considering that these individuals will not only be explicitly instructed to use the MoL but that they will be allowed time for practicing it.

## Methods

The current study was comprised of two experiments. In the first one, episodic memory was assessed with list recall in healthy participants prior to learning MoL. They subsequently learned the technique and completed another list recall task involving never seen before items for which they were instructed to use MoL. The performance comparison between pre- and post-MoL training was used to establish the efficacy of the protocol. In the second experiment, a similar MoL training protocol was adapted to individuals with schizophrenia in terms of duration, sessions, and the number of items. The overall procedure for both experiments is described below; when the procedure diverged among studies, differences were described.

### Participants

Seventy-one healthy participants were recruited through online advertisement (i.e., Kijiji, Facebook groups) and word of mouth for Experiment 1. Inclusion criteria for healthy individuals consisted in: (a) no past diagnosis of schizophrenia spectrum or related psychotic disorders, (b) no family history of a psychotic disorder; (c) age 18 and over, and (d) English proficiency. Seven participants with schizophrenia were recruited from the Center for Personalized Psychological Intervention for Psychosis (Ci3P) at the Douglas Mental Health University Institute (DMHUI) for Experiment 2. Inclusion criteria for individuals with schizophrenia were (a) diagnosis of schizophrenia spectrum or related psychotic disorder, (b) age 18 and over, (c) clinically stable, (d) English or French-proficiency and (e) able to provide informed consent. Exclusion criteria for both groups consisted of having a history of medical or neurological condition that can affect cognition and a family history of hereditary neurological disorders (e.g., Huntington's disease). Two participants dropped out during the study, one due to worsening symptomatology and the other due to scheduling conflicts. Both studies were approved by the DMHUI's Research Ethics Board and all participants signed informed written consent and received monetary compensation for each session.

Schizophrenia diagnoses were confirmed by consulting participants' medical files and diagnostics information provided by the treating psychiatrist of the Douglas Mental Health Institute was retrieved.

### Evaluation Measures

Healthy individuals completed the experiment in one session of 3–4 h duration. Individuals with schizophrenia were offered two sessions of up to 2 h each, on average 1 week apart. All participants filled out a sociodemographic questionnaire that included questions regarding age, current occupation, level of education, gender, and frequency of memory training in daily life. For healthy individuals, questions regarding a past diagnosis of mental disorders, for themselves and family members, were included. Participants with schizophrenia underwent symptoms assessment using the Scale for the Assessment of Negative Symptoms (SANS) (Andreasen, 1989) and the Scale for the Assessment of Positive Symptoms (SAPS) (Andreasen, 1984). Assessments were conducted by trained research staff who previously achieved inter-rater reliability scores in the good to excellent range on the SAPS and SANS (Konsztowicz et al., 2018). Antipsychotic medication doses were obtained from individuals' medical files, and chlorpromazine-equivalent doses were calculated according to Woods (2003).

#### Pre-training Assessment

**Figure 2:**
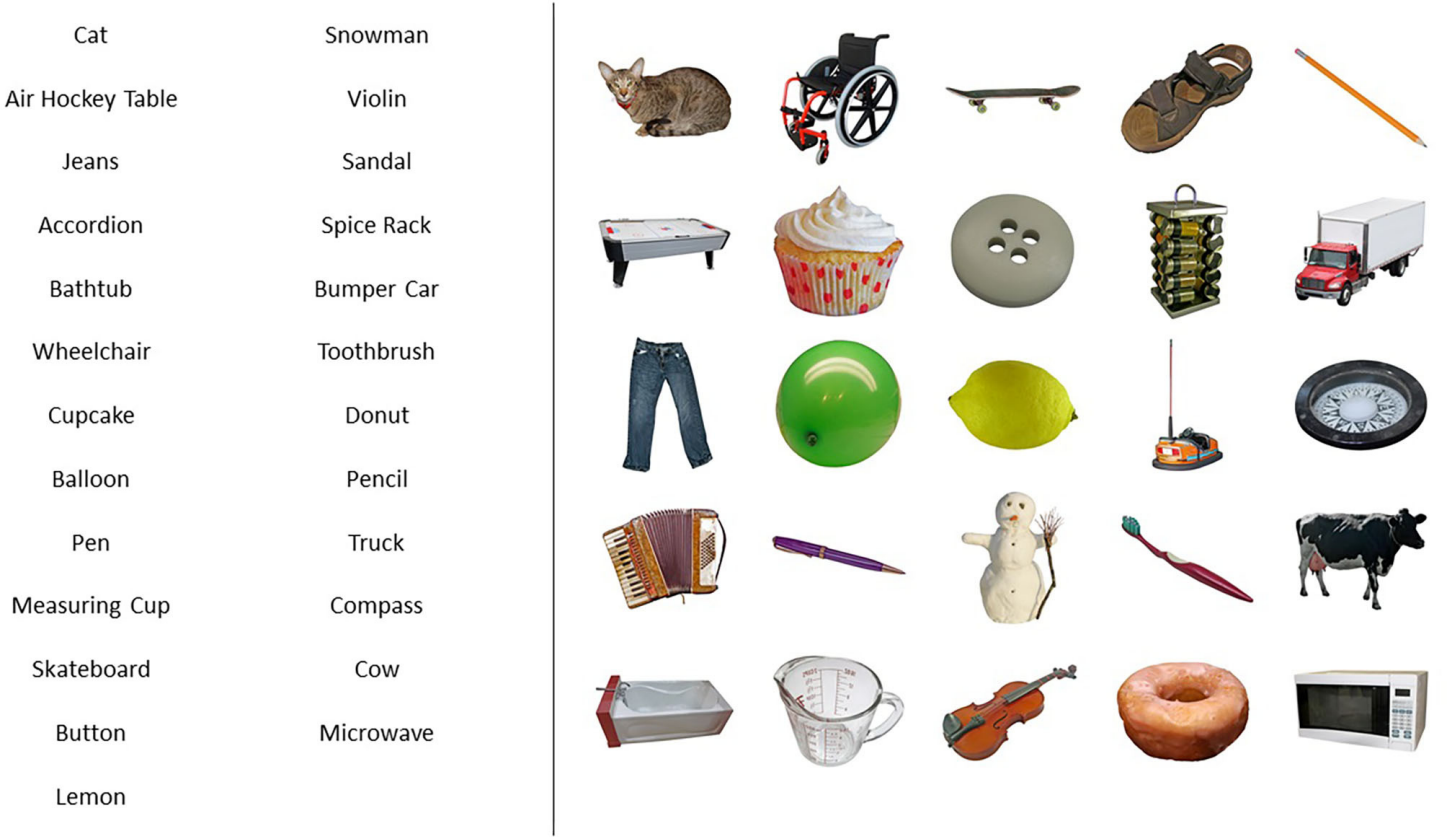
Example of a list of words (left) and a list of images (right) used in the experiments. Lists were 25-items long for healthy individuals and 20-items long for the schizophrenia group. In all the six versions of the memory task (three lists of words, three lists of images), list presentation was alternated so that half of the participants saw 25 or 20 concepts in words and the other 25 or 20 concepts in pictures, and the other half of participants were exposed to the concepts in reverse order.

Healthy participants were asked to learn two 25-item lists, one containing words and the other containing images (photos of single objects) (please refer to Figure 2 for an illustration of the stimuli used). Participants with schizophrenia were asked to learn similar lists but with 20-items each. The shortening of list length was done to decrease overall memory load during loci encoding.

For both groups, each item appeared on a computer screen for 15 s. Following encoding, participants were asked to write down as many items as they could remember, regardless of the order learned (free recall). Participants were asked whether they had used strategies to learn the two lists and if so, to identify them. Subsequently, they completed the Memory Strategy Questionnaire (MSQ) (Hawco et al., 2013), administered to investigate differences in memory strategy use between healthy individuals and those with schizophrenia in daily life.

#### MoL Training and Practice

Participants were given the MoL instructions (based on Bower, 1970's study) and were also provided with examples. Participants were then invited to build a personal “memory palace” using a familiar place as a reference. Help was provided to ensure that the memory palace was following the MoL task requirements. For healthy subjects, that meant 25 loci distributed among five rooms. For individuals with schizophrenia, 20 loci were distributed among four rooms. Loci followed a coherent spatial order that matched the familiar place chosen and were objects/places that stood out in the room and could not be missed (see Figure 1 for details). Participants were asked to recall the loci present in their memory palace without consulting their written list to ensure they could remember the loci and the order of the loci before proceeding to the training lists.

Participants practiced their memory palace by learning two new lists that followed the same parameters used in the pre-training memory task. During practice, they were allowed to make changes in their loci and loci order to improve their memory palaces.

#### Post-training Assessment

In the second session (same session for healthy participants), participants were instructed to use MoL as shown, including writing down their loci next to recalled items. That instruction would later be used to verify the correct implementation of the MoL, by comparing the memory palace's loci used during MoL training and MoL practice to those in the post-training task, however, due to an error in the written instructions, only the loci of remembered items were registered by the majority of healthy individuals.

In all the six versions of the memory task (three lists of words, three lists of images), both the words and the images lists contained different items. Verbal and figural stimuli were included to assess whether MoL efficacy was dependent on the nature of stimuli, as words and images may require different cognitive processes during MoL encoding, due to the visuospatial nature of the task. Images with high name agreement scores were selected from the BOSS database (Brodeur et al., 2010) and their corresponding names were used for the lists of words so that 50 concepts in word version and image version were used in total. Half of the participants saw 25 concepts in words and the other 25 concepts in pictures. The other half of the participants were exposed to the concepts in reverse order. The two groups of 25 concepts were balanced for word agreement according to the BOSS norms (Brodeur et al., 2010). The same procedure was followed with the presentation of the 20-items lists to individuals with schizophrenia.

### Statistical Analysis

Outcome variables included memory performance (number of items recalled pre and post-MoL training); MoL effect (difference in recall pre and post each list type, and total mean recall difference), MoL retention (mean number of loci correctly recalled after training) and MoL use (mean number of loci correctly recalled in the two post-training lists, only for schizophrenia group). MSQ responses were also compared between groups. Statistical tests were performed using IBM SPSS Statistics (version 21 and 26). The normality distribution of the outcome variables was assessed using Shapiro-Wilk tests. In experiment 1, the main outcome variables of MoL effect were not normally distributed (*p*_*s*_ < 0.05), and Wilcoxon Signed Rank Tests were used for the pre and post comparisons. In experiment 2, though the distribution of the outcome variables approached normality, Wilcoxon Signed Rank tests were used due to the small sample size. For memory outcomes results, tests were one-sided, as we assumed improvement in performance after MoL training. For MoL retention and use, tests were two-sided. MSQ responses were compared between groups using Mann-Whitney tests (one-tailed, as we expected individuals with schizophrenia to use fewer strategies than healthy individuals). Effect sizes *r* for non-parametric tests were calculated using the formula *r* = Z/√N as proposed by Rosenthal et al. (1994). Effect sizes *r* between 0.1 and 0.3 are considered small; between 0.3 and 0.5 intermediate, and 0.5 and larger are considered strong (Cohen, 1988). Finally, the mean percentages of answers to the MoL feedback form (administered to individuals with schizophrenia only) were calculated and reported along with the participants' subjective observations.

Supplemental Person's correlation coefficient tests (two-tailed) were conducted to investigate the correlation between age and Mol effect (post- minus pre-test performance) for healthy individuals. For the schizophrenia group, supplemental analyses using the Reliable Change Index (RCI), based on Duff (2012) were performed to assess clinically meaningful change at the individual level, for each list type. RCI's were calculated using the task parameters obtained from the total sample of experiment 1 with non-clinical individuals (standard deviation of pre-test lists and test reliability).

## Results

### Experiment 1: Healthy Participants

In the initial study, 71 non-clinical participants without a family and personal history of psychosis were recruited (see Table 1 for sociodemographic characteristics). Seventy participants completed the pre-post word lists, and 64 completed both words and images lists. Dropout reasons included the extended length of the testing session and fatigue.

**Table 1:** Sociodemographic characteristics of the healthy population sample (*N* = 71), of the schizophrenia recruited sample (*N* = 7) and of the subgroup of schizophrenia completers (*n* = 5).

	**Healthy individuals**			**Schizophrenia (recruited)**			**Schizophrenia (completers)**		
	**Mean (SD)**	**Median**	***N* (%)**	**Mean (SD)**	**Median**	***N* (%)**	**Mean (SD)**	**Median**	***N* (%)**
Gender (Female)			36 (50.7%)			5 (71.4%)			3 (55.6%)
Age (years)	30.3 (10.8)	28		40.8 (13.1)	40		40.8 (11.4)	40	
Education (years)	14.8 (1.7)	15		13.7 (2.2)	13		14.6 (1.7)	15	
MSQ *Items related*	5.2 (1.8)	5.0		3.1 (1.2)	3.0		3.6 (0.9)	3.0	
MSQ *Objects interacting*	4.0 (2.3)	5.0		2.9 (2.1)	4.0		3.8 (1.6)	4.0	
MSQ *Personal memories*	3.8 (2.2)	4.0		4.4 (2.6)	5.0		4.8 (1.8)	5.0	
MSQ *Making sentences*	2.7 (2.2)	2.0		3.0 (2.0)	2.0		3.8 (1.8)	4.0	
MSQ *Repetition*	5.0 (2.2)	6.0		5.6 (2.7)	7.0		5.0 (3.1)	7.0	
*Schizophrenia* diagnosis	–	–				5 (71.4%)			4 (80%)
Other diagnosis	–	–				2^a^ (28.6%)			1^b^ (10%)
Age of onset (years)	–	–		20.8 (7.2)	20		20.8 (8.0)	19	
Duration of illness (years)	–	–		17.2^c^ (14.9)	14		20.0 (14.9)	18	
Chlorpromazine equivalent (mgs)^d^	–	–		616.3 (666.4)	350		742.8 (768.8)	350	
SAPS total	–	–		27.6 (24.2)	18		31.0 (28.5)	18	
SANS total	–	–		25.1 (7.5)	27		22.8 (7.5)	23	

*MSQ, Memory Strategies Questionnaire; SAPS, Scale for the Assessment of Positive Symptoms total (composite) score was calculated by summing all items except for the global rating items; SANS, Scale for the Assessment of Negative Symptoms total (composite) score was calculated by summing all items except for the global rating items; MSQ, Memory Strategies Questionnaire. Other diagnosis included schizoaffective disorder^a.b^ and bipolar disorder with psychotic features^a^*.

c *N = 6. Data was missing for one participant that refused access to medical file*;

d *Antipsychotic chlorpromazine-equivalent dose was calculated according to Woods (2003)*.

#### Strategy Use Prior to MoL Training

When asked whether and subsequently which strategies they used to learn items in the pre-test, on average 56% of participants reported as primary strategy making sentences or making a song using list items, 32% imagined items interacting in some way (i.e., making a story, linking items together), 23% considered how objects were related to each other (i.e., categorizing or grouping similar items), 13% associated items to personal memories, 13% used repetition, and 13% used other strategies, such as visualizing the items, memorizing the first letter of the item's name, organizing items in alphabetical order, making an acronym or grouping objects into one or more rooms.

The MSQ, a general measure of strategy use in real life was administered to all participants and the most often used strategies endorsed were “items related” (“I considered how the items could be related to each other”), and “repetition” (“I repeated the name of the items to myself in my head”). These were followed by “objects interacting” (“I imagined the items interacting in some way”) and “personal memories” (“I used prior personal memories associated with the items”). The least used strategy was “making sentences” (“I constructed a sentence with the two items”) (see Table 1 for descriptive statistics).

#### MoL Learning and Use

MoL retention was verified during MoL training by asking participants to recall their personal list of 25 loci (to be used during the experiment) without consulting their written list. The mean number of loci recalled during MoL training was 24.68 [standard deviation (*SD*) = 0.91] (total) and 20.30 (*SD* = 6.95) (correct order). During the experiment sessions, it was observed that some participants were not compliant with MoL instructions and did not use MoL as shown during the post-test. MoL compliance was later verified for each participant by comparing their “memory palace sheet” (list of loci) made during training to the loci reported next to items in the two post-training lists, and by participants' admissions of not using MoL in the post-training lists. Ten participants (14.1%) seemed to not be using MoL at post-training. Participants using MoL were significantly younger than those not using MoL [mean difference (*MD*) = 10.4 years, *SE* = 3.5, *p* = 0.004].

#### Memory Outcomes

**Figure 3:**
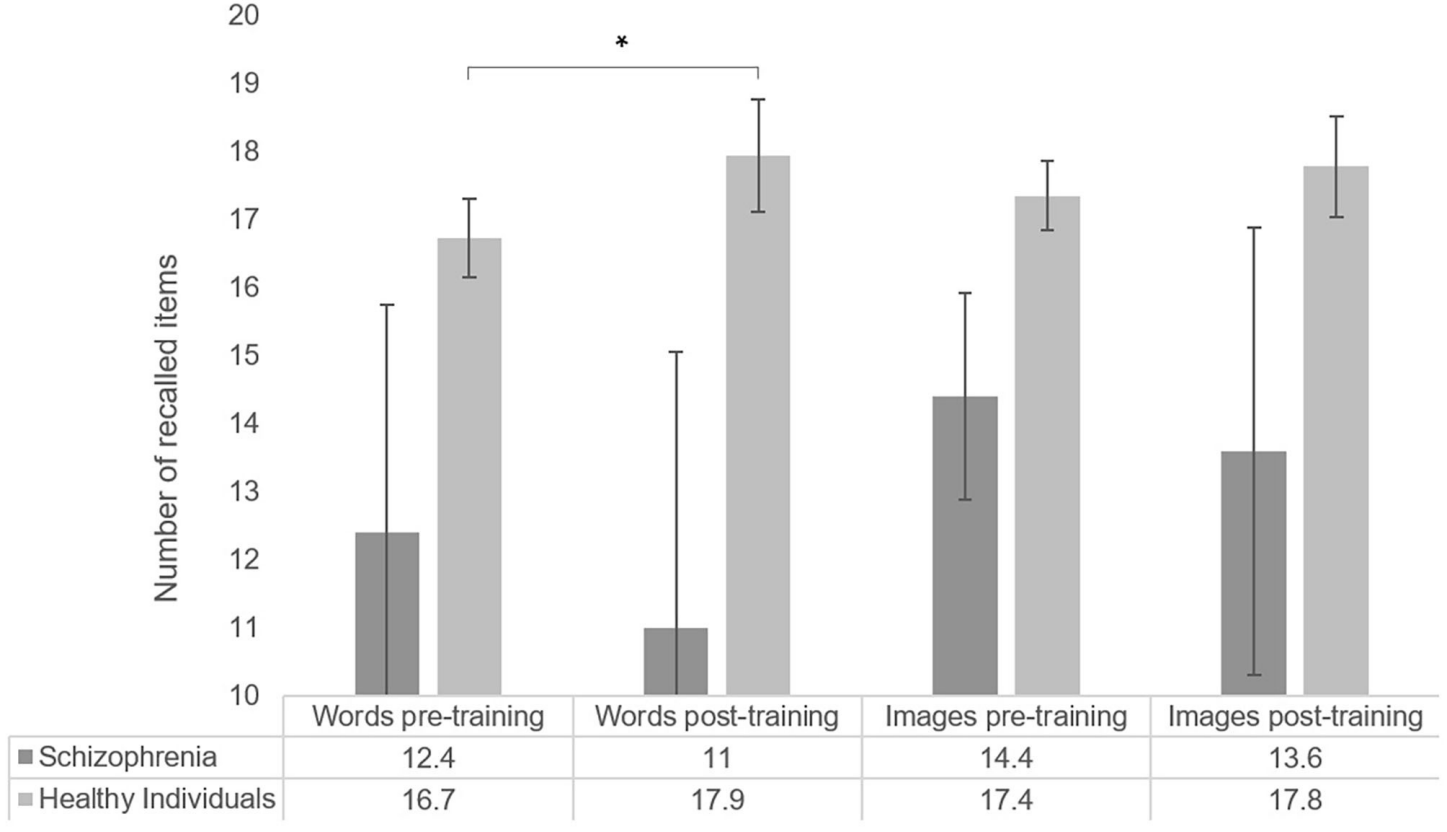
Number of items recalled by list type, for healthy participants (word list *N* = 70; image list *N* = 64) and individuals with schizophrenia (*N* = 5). Error bars represent standard error of the mean. *p < 0.05.

The average number of items recalled by list type and overall are shown in Figure 3. Pre-training median recall (median number of items recalled, including recall of similar items, in both lists) was 17.5 items, and post-training median recall was 19 items.

When comparing pre-post list performance, we observed a significant improvement in the list of words (*T* = −1.72, *p* = 0.04, *r* = −0.15, one-sided), while performance in the list of images did not significantly differ after MoL training (*T* = −0.35, *p* = 0.363, *r* = −0.03). Pearson's correlation coefficients suggested no association between age and Mol effect for words (*r* = −0.09, *p* = 0.45) nor for images (*r* = −0.16, *p* = 0.22).

After excluding non-compliant participants (*N* = 10), pre-training median recall was 18 items and post-training median recall was 19.75 items.

When comparing pre-post list performances among compliant participants, we found a significant overall improvement after MoL training (*T* = −2.22, *p* = 0.013, *r* = −0.20) that was driven by a significant and larger improvement in the list of words (*T* = −2.91, *p* = 0.002, *r* = −0.30), while performance in the list of images was again not significantly different after MoL training (*T* = −1.52, *p* = 0.065, *r* = −0.14). Pearson's correlation analyses failed to show a significant association between age and Mol effect among compliant participants (words *r* = −0.24, *p* = 0.85, images *r* = −0.14, *p* = 0.31).

### Experiment 2: Individuals With Schizophrenia

Five individuals with schizophrenia completed MoL training (see Table 1 for their sociodemographic and clinical characteristics).

#### Strategy Use Prior to MoL Training

Individuals with schizophrenia were asked which strategies they used to learn the items. Four participants mentioned the sparse use of at least one strategy, applied only to certain items in each list rather than consistently across both lists. Strategies included categorizing, making relationships with familiar things, associating with personal experiences, translating, visualizing, rehearsing, making sentences, making a song with the words, and making a narrative. In the MSQ, the most often used strategy reported was “repetition” (“I repeated the name of the items to myself in my head”), followed by “personal memories” (“I used prior personal memories associated with the items”), “items related” (“I considered how the items could be related to each other”), and “making sentences” (“I constructed a sentence with the two items”). The least used strategy was “objects interacting” (“I imagined the items interacting in some way”) (see Table 1 for means and SDs). Group comparison suggested individuals with schizophrenia reported using significantly less “item-related strategies” (*U* = 88.5, *p* < 0.01, *r* = −0.03, one-tailed) and tended to use less “objects interacting” (*U* = 167, *p* = 0.08, *r* = 0.02, one-tailed) in the MSQ compared to healthy individuals.

#### MoL Learning and Use

**Figure 4:**
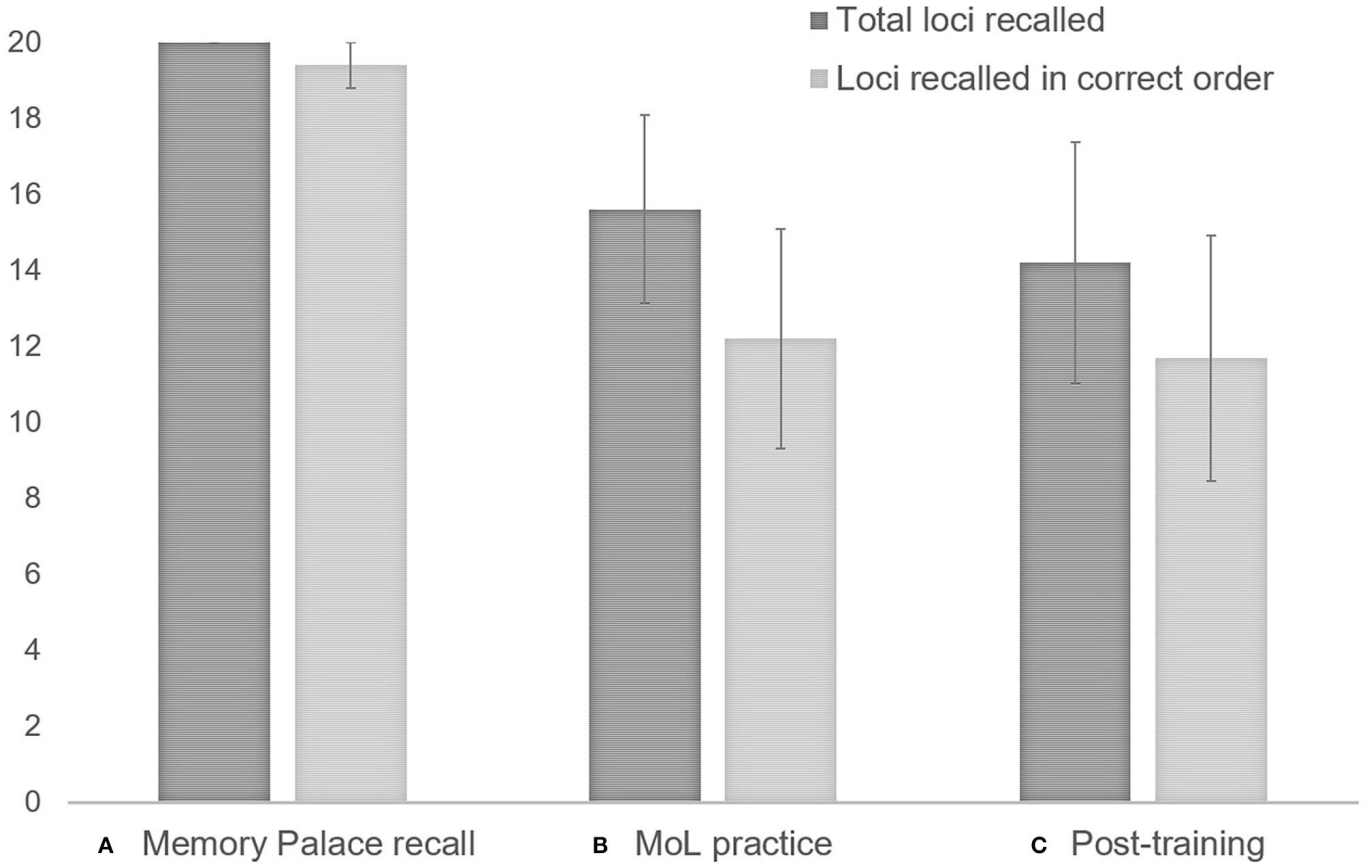
Individuals with schizophrenia use of MoL: **(A)** Memory palace recall (participants were asked to recall loci without looking at the Memory Palace sheet); **(B)** MoL practice: mean number of loci recalled (total and in correct order) for the two^1^ practice lists; **(C)** Post-training recall: mean number of loci recalled (total and in correct order) for the two^1^ final lists. ^1^When participants did not report loci in one of the two lists, only loci for the list reported were computed. Error bars represent standard error of the mean.

MoL learning of individuals with schizophrenia was determined by the mean number of loci correctly recalled (total and in correct order) in the two practice lists. MoL use was quantified by the mean number of loci correctly recalled (total and correct order) in the two post-training lists. The mean number of loci recalled (both total and in the correct order) during MoL training tended to be lower than during memory palace loci recall (total: *T* = −2.02, *p* = 0.06, *r* = −0.6; correct order: *T* = −2.02, *p* = 0.06, *r* = −0.6). No other significant differences or trends between means were observed across time-points. The results are illustrated in Figure 4.

#### Memory Outcomes

The total median recall of individuals with schizophrenia in the pre-training was 13.5 items (13 words and 15 images) and the post-training median was 12.5 (13 words and 12 images). No significant effect of MoL on recall was observed in the pre-post memory performance comparison (*T* = −0.68, *p* = 0.313, *r* = −0.2). The means of pre-and post-training recall for the list of words and the list of images also did not significantly differ (words: *T* = −0.41, *p* = 0.406, *r* = −0.1; images: *T* = −0.962, *p* = 0.250, *r* = −0.3). Recall performances are shown in Figure 3.

RCI analyses of patients' individual scores suggested no significant change for the majority of participants in either list (*p*_*s*_ < 0.05), except for one participant that performed significantly worst in the word list after MoL training (RCI *z*-score = −1.83, *p* < 0.05). Participants RCI *z*-scores in each list were reported in Table 2.

**Table 2:** Recall discrepancies (RD) and Reliable Change Index (RCI) *z*-scores for schizophrenia participants by list type.

	**Schizophrenia participants**				
	**P1**	**P2**	**P3**	**P4**	**P5**
Word list RD	6	3	−2	−9	−5
RCI	1.22	0.61	−0.41	−1.83^*^	−1.01
Image list RD	−2	1	−3	−2	2
RCI	0.41	0.21	−0.62	−0.41	0.41

*RD is calculated by subtracting the number of items recalled at pre-training from that after MoL training, so that negative values indicate performance improvement. RCI z-scores are compared with a normal distribution table and indicate “no change” between pre- and post-training scores when falling above or below the typical cut-off of ±1.645 for a 5% confidence level (Duff, 2012)*.

* *p < 0.05*.

#### Individuals With Schizophrenia's Appraisal of Intervention

Feedback from individuals with schizophrenia was obtained using a post-intervention questionnaire comprising five yes/no questions. Questions included whether they had used and modified MoL and whether they had difficulty learning and using MoL. All five participants reported using the MoL as learned during training. Three out of the five participants reported having difficulty learning MoL, and four reported having difficulty using MoL. When asked if they would consider using MoL on a day-to-day basis, four answered “yes”.

Participants were asked to provide further explanations if they had answered having difficulty learning or using MoL. All but one reported having difficulty remembering their loci or their loci order during the post-training, which might have interfered in their ability to use the MoL correctly.

Regarding specific difficulties learning MoL, participants mentioned that MoL required a lot of energy to concentrate and to “*make the brain works;”* that the order of the loci was difficult to learn and remember, and that making the associations [between the loci and items] “*big and weird”* as suggested during training was difficult.

Regarding difficulties using MoL, participants reported that challenges included fatigue, lack of familiarity with the technique, and the concept that the order of the items related to the order of the loci was difficult to follow. One participant recognized that the practice helped with using MoL and that using it with images was easier than with words.

## Discussion

We investigated the application of a known mnemonic, the Method of Loci, as part of an intervention to improve episodic memory performance in non-clinical subjects and schizophrenia. The intervention protocol was initially tested in a first experiment with healthy individuals, for which memory performance was compared before and after training using two types of stimuli (words and images). We observed significant improvement restricted to words recall. In a second experiment, we adapted the parameters of MoL for individuals with schizophrenia by decreasing the number of items in each list, doubling the number of sessions and decreasing session duration. Individuals with schizophrenia failed to improve with MoL, despite successful comprehension of technique at training. The examination of case by case failed to reveal any individual with schizophrenia who significantly improved with the technique. Hence, we suggest that MoL may not represent a promising strategy to improve memory in schizophrenia and we discuss below the reasons why.

### MoL Effectiveness and Acceptability in Healthy Individuals

In healthy individuals, MoL was well-tolerated for the majority of participants (86%). MoL compliant participants were on average 10 years younger than the non-compliant, accompanying previous evidence that younger adults are more likely to comply with MoL and follow instructions correctly (Verhaeghen and Marcoen, 1996).

A training protocol based on previous literature was developed to teach the Method of Loci, and different list types were used to assess whether MoL's effectiveness was dependent on the nature of stimuli, as words and images may require different cognitive processes during MoL encoding. Results suggest that MoL's effectiveness was restricted to the list of words, as no improvement was found in recall performance of the list of images. We hypothesize that MoL requirement for mental manipulation of stimuli to incorporate it to loci requires more processing for word stimuli than for images. Incorporating images to loci “as seen,” therefore, resulting in a weaker memory trace.

The effect size of the pre-post memory improvement found in the list of words was in the small range, in disagreement with previous reports of Loci effects in the general population. For instance, a review of several studies comparing the effects of memory training in younger and older adults using MoL (Verhaeghen and Marcoen, 1996) found effect sizes *d* between 0.8 and 1.85 for older adults, and up to 6.59 among younger MoL users. The small improvement with MoL in our sample could be a reflex of insufficient training or participants' fatigue at the end of the experiment, which prevented optimal encoding of the last two lists. Though we did not control for the last, fatigue was subjectively observed by the experimenters and frequently reported by participants at the end of the sessions. In an attempt to diminish the effects of fatigue in memory performance in our subsequent study with individuals with schizophrenia, we split the experiment into two sessions of half the length in experiment 2. We also included extra instructions and guided encoding during practice lists to ensure understanding of the MoL technique prior to testing.

### MoL and Schizophrenia

Overall, individuals with schizophrenia understood the MoL and had no difficulty making a Memory Palace, which was based on their homes in all cases. During MoL training and practice (“memory palace recall” and “practice lists”), participants showed acceptable retention of their chosen loci and loci order. Despite overall good feedback to MoL, they expressed difficulty using MoL to encode new lists, which ultimately prevented memory performance improvement.

Individuals with schizophrenia included in this intervention had wide variability in positive symptoms scores and a more similar range of negative symptoms. The majority of participants in this sample reported self-generating strategies at the pre-training, some involving shallow semantic processing (i.e., translating, rehearsing, visualizing) and some involving deep semantic processing (i.e., categorizing, making sentences), associative strategies (i.e., forming relationships among familiar things, associating with personal experiences) or both (i.e., making a song with the words, making a narrative). However, strategies were applied sparsely rather than consistently throughout the lists, which might explain their low efficacy in our sample.

When asked about their general use of strategies in daily life, participants in the schizophrenia group reported using shallow processing strategies more often than associative (e.g., linking words together, making sentences) and semantic strategies. Furthermore, the frequency of associative/semantic strategies such as relating items to each other and imagining items interacting with each other was inferior compared to healthy controls. This is in accordance with the literature reporting strategy use in the schizophrenia population (Koh et al., 1973; Koh and Peterson, 1978; Gold et al., 1991; Paulsen et al., 1995; Iddon et al., 1998; Hazlett et al., 2000; Brébion et al., 2004b; Gsottschneider et al., 2011).

Individuals with schizophrenia expressed difficulty creating relationships between the loci and items, and keeping track of the loci and loci order, during encoding of new lists. These activities rely heavily on the use of executive functions and speed of processing dimensions known to be affected by schizophrenia (Heinrichs and Zakzanis, 1998; Flashman and Green, 2004; Schaefer et al., 2013). These results are also in keeping with previous findings in which individuals with schizophrenia might benefit less than non-clinical controls from visuospatial mnemonic strategies (Iddon et al., 1998).

Both factors might have influenced MoL use in our study, explaining the lack of improvement after training. Other factors that may have interfered with the correct use of MoL were fatigue (that persisted despite the decrease of session duration), lack of creativity to form salient relationships between items and loci, and interference of previous lists when learning new lists.

Many of the challenges we faced teaching MoL to individuals with schizophrenia is in line with reflections on the use and efficacy of imagery mnemonics in memory remediation for individuals for which memory is impaired as a result of brain injury or disease, provided by Richardson (1995). According to Richardson, although training in mental imagery (i.e., the pegword method and the method of loci) led to improved performance in some brain damage patients, improvement was largely dependent not only on the etymology of the damage (and consequent severity of memory impairment), but in patient's motivation, preservation of mental imagery capabilities, imagination, awareness of memory deficit, and the need of explicit prompting for successful use of mental imagery at recall. Finally, and of high similarity to our observations, improvement in such individuals was often unneglectable (i.e., a gain of on average one item as a result of several training sessions), therefore not compensating the costs and time associated with delivering this training as part of a cognitive remediation program.

### Limitations

The current feasibility study has several limitations. First, our experiment with non-clinical participants did not include a control group. As such, it is not possible to rule out the presence of a practice effect in the memory improvement found after MoL training. Second, non-clinical and schizophrenia groups were not matched on age. Considering previous evidence that older individuals tend to be less compliant to MoL and have less cognitive flexibility to adopt new strategies (Verhaeghen and Marcoen, 1996), age could potentially have been a confounder in our group comparisons as patients were on average 10 years older than the healthy individuals included in the study. However, additional analyses suggested no significant association between age and MoL effect in non-clinical participants, both for the entire sample and when excluding non-collaborative participants. Third, our sample size of individuals with schizophrenia was small, reducing the power of our analyses and comparisons to the healthy control group. Moreover, this small sample size made it impossible to control for antipsychotic medication and chronicity of illness. Considering the apparent lack of beneficial effect of MoL on performance combined with the apparent high cognitive demands of MoL in individuals with schizophrenia, there seemed to be very few advantages in pursuing recruitment. Fourth, we did not include in our assessment protocol a measure of depressive symptomatology nor one for current drug use in our study. Both factors are known to affect memory performance (Brebion et al., 1997; Brébion et al., 2004a). Finally, the suboptimal parameters of training in non-clinical individuals (i.e., fatigue and insufficient training) may have contributed to the lack of significant findings in schizophrenia. Although we modified such parameters to adapt the intervention with clinical participants, they may not have been sufficient. Considering all these different limitations, our results should be interpreted and generalized with caution.

## Conclusions

Beyond those limitations, the null results combined with our experience conducting this study suggest that MoL may not be an ideal strategy to implement in a cognitive remediation therapy to improve episodic memory in schizophrenia. The high cognitive demand and restricted applicability of MoL in contexts other than list learning would make this strategy difficult to implement in a program whose ultimate goal is to reflect functional gains. Future research should consider expanding on strategies already proven efficient in schizophrenia, such as the Strategy for Semantic Association Memory (SESAME) to improve self-initiation of semantic encoding (Guimond et al., 2018), and should investigate simpler strategies [e.g., unitization (D'Angelo et al., 2015, 2016)], which are effective in populations with similar severity of cognitive deficits such as those found in schizophrenia.

## Data Availability Statement

The raw data supporting the conclusions of this article is not available due to ethical restrictions.

## Ethics Statement

The studies involving human participants were reviewed and approved by Douglas Mental Health University Institute Research Ethics Board. The patients/participants provided their written informed consent to participate in this study.

## Author Contributions

AES contributed with data study designing, data collection, statistical analysis, and manuscript writing. YM contributed with data collection, data analysis, and revisions. MB contributed with study design, data analysis, and revisions. ML contributed with data analysis, writing, and revisions. All authors contributed to the article and approved the submitted version.

## Conflict of Interest

The authors declare that the research was conducted in the absence of any commercial or financial relationships that could be construed as a potential conflict of interest.

## Appendix

A literature search on the Medline (Ovid) database from inception to December 2020 using a combination of the Medical Subject Headings (MeSH) and search terms “schizophren*” OR “schizo-affective” OR “psychos*” OR “psychot*” -AND “method of loci,” -AND “mnemonic*”; -AND “memory palace” failed to identify any studies with MoL in schizophrenia.
